# Adaptive production strategy in vertical farm digital twins with Q-learning algorithms

**DOI:** 10.1038/s41598-025-97123-y

**Published:** 2025-04-30

**Authors:** Yujia Luo, Peter Ball

**Affiliations:** 1 School of Business and Society, The University of York, York, United Kingdom; 2 York Business School, York St John University, York, United Kingdom

**Keywords:** Digital twin, Urban food system, Adaptive production strategies, Q-learning network, Engineering, Mathematics and computing

## Abstract

Urban food production can contribute to sustainable development goals by reducing land use and shortening transportation distances. Despite its advantages, the implementation of digital twin (DT) technology for urban food systems has received less investigation compared to manufacturing. This article examines the influence of DT technology on adaptive decision-making in urban food production, focusing on the “Grow It York” case study. Utilising mixed integer linear programming (MILP) and Q-learning models, this study explores how DT data enhances production decisions regarding service level and resource utilisation under demand fluctuations. The findings highlight that the Q-learning model achieves up to $78.5\%$ demand fulfillment compared to $58.5\%$ for the MILP model, demonstrating a significant improvement in operational efficiency. Additionally, electricity usage per fulfilled demand is reduced by approximately $15\%$, advocating for broader DT application to the synergy between economic resilience and environmental sustainability. Future research directions include scaling DT implementation to manage complex supply chains, including advancing real-time data integration and incorporating sustainability considerations at supply chain level.

## Introduction

The intensification of urbanisation and climate change poses significant challenges to global food security, prompting the exploration of innovative agricultural solutions such as urban vertical farming. Utilising technologies like hydroponics and aeroponics, urban vertical farms capitalise on underused spaces (e.g., rooftops and basements) to bring food production closer to consumption centres^1^. This approach not only optimises land use but also minimises the reliance on extensive rural farmland and reduces transportation-related emissions^2^. Research into smart urban food systems is gaining traction, with recent discussions focusing on how digital twin (DT) technology can be adapted from traditional manufacturing to increase efficiency and digitalisation in urban agriculture (Agriculture 4.0). A DT is a virtual representation of a physical system that is updated with real-time data to mirror and predict the performance and behaviours of the physical counterpart^3^. Despite the potential of DT technology in urban food production, significant research gaps remain. Existing studies have primarily focused on the theoretical potential of DTs, with limited empirical evidence on their practical implementation and effectiveness in real-world settings^4^. Moreover, there is a scarcity of research on how DTs can be integrated with advanced optimisation models to handle the complexities of demand uncertainty and resource constraints in urban vertical farms. Additionally, the application of reinforcement learning techniques, such as Q-learning, to enhance adaptive decision-making in DT-driven production scheduling has not been thoroughly explored. This study addresses these gaps by providing empirical evidence of the benefits of DT integration with Q-learning models in improving demand fulfillment and resource efficiency in urban vertical farming. However, the unique attributes of urban farming mean that direct application of DT technology from manufacturing may not always be appropriate.

In the context of vertical aeroponics farms, the basic structural and operational components are core to ensure the system’s functionality, such as growth towers, nutrient delivery systems, and environmental control units^1^.The process flow, level of monitoring and control and the level of technology deployment mean such farming is conceptually close to typical production systems. The farming process steps that follow planting, such as nutrient delivery and environmental control, often vary and can lead to hybrid operations combining automated and manual interventions. The supply and maintenance of these core components are critical and often unpredictable in terms of performance and wear. The quality of grown plants is regularly related to optimal controlled environmental conditions, but its classification and monitoring are often performed manually or with basic automation, which may not fully utilise the potential of smart technologies. Additionally, the demand for fresh product from urban farms is also volatile. Variability in supply and demand creates complex business models and production scheduling that require flexibility, which until recently, could only be managed manually^5^.

However, as DT technology advances and become more accessible, new opportunities arise to improve the management of uncertainties by providing near real-time information about crop performance, predicting optimal harvest times and conditions, and autonomously adjusting the growing environment to meet multidimensional needs of the crops and the business^6^^7^. The unique attributes of urban food production, such as the use of controlled environment and advanced farming techniques, make DT integration ideal. This sector has not exploited DTs to the same extent as traditional manufacturing. In this context, the advent of DT offers transformative potential for urban food production systems. DTs-dynamic virtual models of physical systems-enhance decision-making by providing insights into operations dynamics^8^.

This study focuses on the “Grow It York” case study to investigate the application of DTs in urban food production, with a particular emphasis on optimal production scheduling and demand fulfillment. By integrating mixed integer linear programming (MILP) and Q-learning models, this research explores how real-time data signalling through DT can optimise decisions related to demand fulfillment and resource efficiency. These advanced models allow for precise adjustments in the growing environment, leading to improved crop yields, better alignment with demand, and more efficient resource management. The findings demonstrate that DT technology can markedly improve operational efficiency and robustness, underscoring the need for broader adoption in food production systems to boost resilience and sustainability.

Following a literature review on DT technology and its applications in urban food production, this paper provides a detailed description of the employed MILP and Q-learning models. The results section presents the findings from the “Grow It York” case study, highlighting the improvements in resource efficiency, and demand fulfillment. The discussion section interprets these results in the context of existing literature, responding to the benefits and challenges of implementing DT technology in urban food production. The conclusion summarises the key insights and suggests directions for future research, including scaling models to handle complex DT data and incorporating environmental and social impact considerations.

## Literature review

The domain application field of DTs is manufacturing, and there is limited research looking at applying DT for modelling biological or flow processes or systems such as agriculture. Current DT applications in agriculture are emerging from creating DTs for crops, farm units, or cultivated landscapes. Kampker et al.^9^ explore the creation of a “Digital Potato” as a smart service for optimising potato harvester calibration using sensors and machine learning to minimise damage and ensure efficiency. Skobelev et al.^10^ extend this by developing a DT for wheat using a multi-agent system and a comprehensive knowledge base to address the limitations of traditional data-driven models, particularly under changing climatic conditions. Laryukhin et al.^11^ propose a cyber-physical multi-agent approach to manage the complexity of agricultural systems. This involves entities such as soil, fertilizer, and crops, represented as agents within a virtual market, optimising resource use and cost. Kim and Heo^12^ applied DT for mandarins farms to visualise and analyse at regional, inter-orchard, and intra-orchard scales and used machine learning to predict performance output (e.g., sugar content and fruit size). While these approaches enhance localised decision-making, they often focus narrowly on crop-specific monitoring, lacking the integration of adaptive decision-making capabilities across broader agricultural systems.

Adaptive decision-making frameworks in agriculture are critical for addressing resource and demand variability. A promising approach integrates simulation modelling, machine learning, and advanced algorithms, outperforming traditional production scheduling by improving the delivery of products and services, especially during disruptive events^13^. Traditional optimisation techniques, such as MILP, are foundational in production scheduling due to their ability to model constraints and allocate resources efficiently. For example, Ghandar et al.^2^ successfully applied advanced DT production planning across different scales in a regional aquaponic farm system. Li et al.^14^ conducted a system modelling of a vertical farm in Singapore regarding crop yield, energy, and energy flow, and sustainability assessment into a mixed integer nonlinear programming (MINLP) optimisation model to maximise the economic profits. In food supply chain contexts, Maheshwari et al.^15^ and Corsini et al.^13^ demonstrated MILP’s capacity to optimise procurement, production, and distribution under constraints. However, its static nature and computational demands under dynamic and uncertain conditions highlight its limitations.

Q-learning offers adaptability by learning from evolving environmental conditions, excelling in dynamic decision-making scenarios^16^. However, its computational intensity and need for extensive training data present challenges. Research by Du et al.^17^ emphasises combining reinforcement learning with optimisation models to enhance performance in environments characterised by uncertainty and variability. To address the limitations of standalone methods, hybrid approaches integrating MILP, simulation, and machine learning are gaining traction. Badakhshan and Ball^5^ presented a hybrid framework combining discrete-event simulation, optimisation, and machine learning for supply chain disruptions. Similarly, Maheshwari et al.^15^ utilised agent-based simulations with optimisation to improve scheduling and reduce disruptions in food supply chains. These frameworks demonstrate significant improvements in adaptability and computational efficiency, paving the way for their adoption in agricultural systems.

Recent advancements have begun to address interdisciplinary gaps, focusing on integrated models for farm-level optimisation. For example, Li et al.^14^ employed system modeling and optimisation for vertical farms, integrating sustainability metrics into economic optimisation. Their model, although effective, lacked real-time adaptability, a limitation addressed by dynamic DT frameworks. Rosario Corsini et al.^13^ highlighted the potential of DTs to integrate real-time monitoring with adaptive decision-making, ensuring resilience in production-distribution systems under disruptions.

While prior research has demonstrated the utility of MILP for resource allocation in urban farming^14^ and the application of DTs in aquaponics for enhanced monitoring^2^, these methods remain limited by their reliance on static optimisation and lack of real-time adaptability. Efforts in food supply chains have achieved significant progress in real-time monitoring and control but often overlook adaptive decision-making and real-time customer order prioritisation^15^. Similarly, energy efficiency improvements in vertical farming have highlighted sustainability challenges but fail to dynamically incorporate energy costs into optimisation processes^1^. To address these gaps, this study introduces a hybrid MILP-Q-learning digital twin framework specifically tailored for urban farming systems, responding effectively to the identified need for DT applications in agriculture^4^. This approach integrates robust optimisation with dynamic adaptability, enabling real-time demand fulfillment, energy-efficient resource allocation, and enhanced scalability. By actively responding to changing environmental and operational conditions, the proposed framework outperforms previous methods that primarily focus on monitoring or static optimisation, demonstrating superior performance in meeting demand fluctuations and reducing energy costs. Table 1 presents a summary of the related studies regarding research focus and methods deployed.

**Table 1:** Summary of relevant literature.

Research Stream	Reference	Application context	Focus	Hybrid Modeling Approach
Dt in agriculture	Kampket et al.^9^	Potato harvesting	Business model of potato DT	NA
	Skobelev et al.^10^	Wheat farming	Multi-agent DT ontology for wheat growth and yield forecast	Agent-based simulation and traditional optimisation
	Kim and Heo^12^	Mandarin orchards	Multi-scale DTs of orchards for sugar content and fruit size prediction	Automated machine learing algorithm
	Li et al.^14^	Vertical farming	MINLP optimisation with sustainability assessment; system modeling	MINLP optimisation
	Ghandaret al.^2^	Aquaponic systems	Fish and plant growth predicton	Comparisons among Linear regression, support vector regression, decision trees, XGBoost with decision trees
DT in production and supply chains	Badakhshan & Ball^5^	Supply chain planning	Decision-making under disruptions	Discrete-event simulation and decision-tree algorithm
	Corsiniet al.^13^	Manufacturing supply chain	Replenishment and storage resilience under disruptions	Artificial neural network and particle swarm optimisation
	Maheswariet al.^15^	Food supply chain	Supply chain productivity	Agent-based simulation and MILP
	Du et al.^17^	Flow shop scheduling	Assembly completion time and energy efficiency	Knowledge-based bi-objective collaborative optimisation and Q learning
	**Current Study**	Urban vertical farming	DT implementation for yield with demand fluctuation and energy consideration	MILP and Q-learning algorithms

## Case description

Grow It York is a vertical aeroponic farm where plants are grown in a soil-free environment and nutrients are delivered via a mist^18^. This study focuses on leveraging DT technology to enhance proactive and adaptive production modelling with the case study of Grow It York. By investigating the DT configuration and data integration scheme of the farm, we aim to demonstrate the value of DT data for optimising production scheduling.

**Fig. 1:**
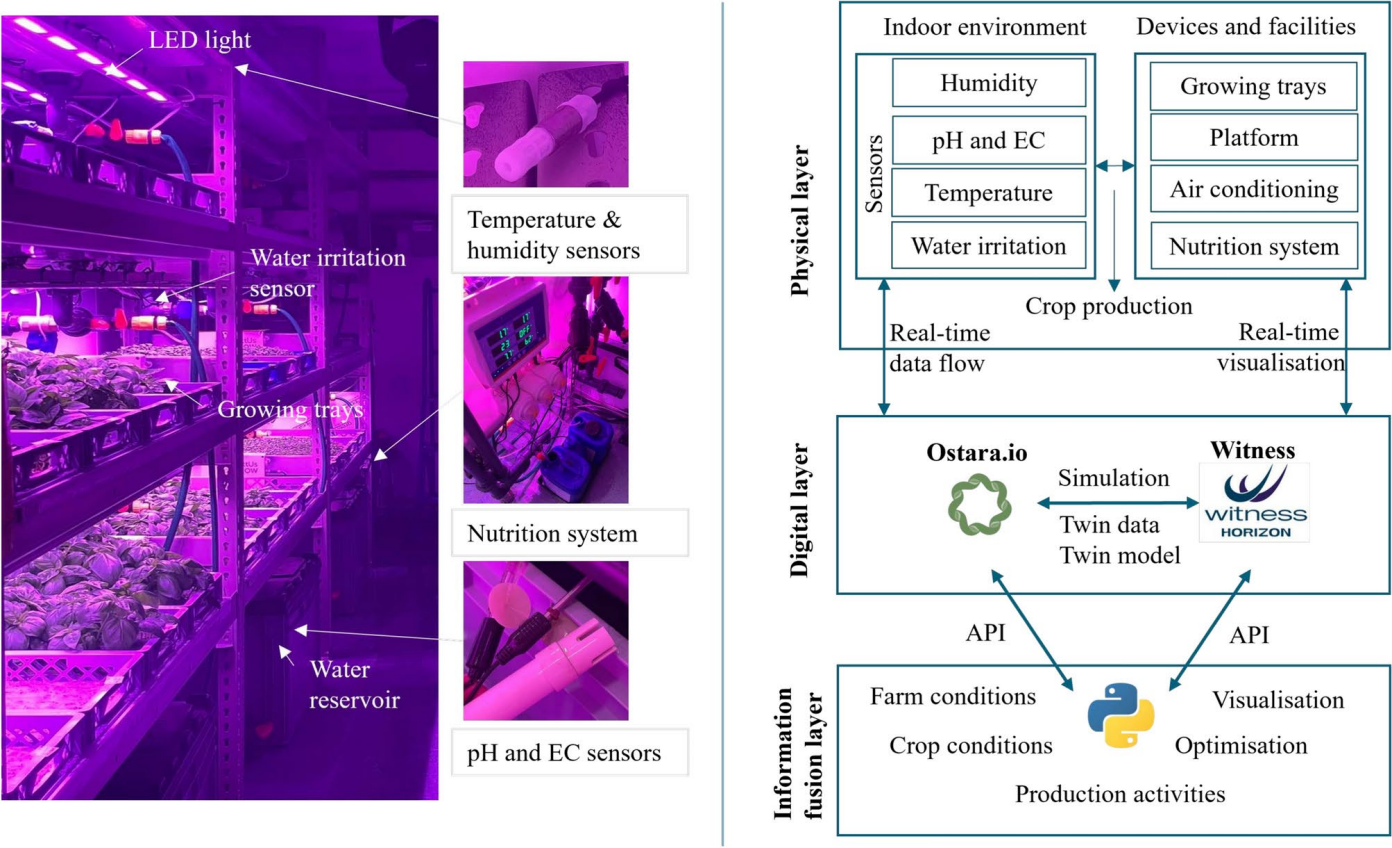
Digital twin configuration in the vertical farm.

Figure 1 presents the physical farm and its DT configuration, including digital layer and information fusion layer. The physical farm maintains a controlled indoor environment to create optimal growing conditions for various crops. The physical setup ( see the left part of Figure 1) includes vertically stacked growing trays, LED lighting systems, environmental monitor units, and a central nutrient delivery system. Sensors, including temperature and humidity sensors, pH and electrical conductivity (EC) sensors, and irrigation sensors, are used to monitor the indoor environment and irrigation schedules. Sensor data is collected and used to monitor crop growth in the operating system Ostara, which is technically supported by LettUs Grow ltd.^19^.

The digital layer represents the virtual farm through the Ostara platform and the simulation software Witness Horizon (Witness). Ostara and Witness mirror real-time data flows and production activities, enabling what-if simulations based on predefined crop production scenarios. Witness complements the Ostara system with its visualisation and simulation functions. The information fusion layer acts as an analysis engine for the farm’s DTs, where real-time DT data related to crops, the indoor environment, production operations, and supply chains are integrated and synchronised for production optimisation. Real-time optimal solutions generated in the information layer using Python (a programming language) update simulation scenarios and estimations in Ostara and Witness for real-time monitoring and adaptation. The bidirectional data automation between the physical and virtual layers, and between the virtual and information layers, is essential for monitoring farm operations. APIs among Ostara, Witness, and Python ensure seamless data exchanges.

DT data automation can facilitate the estimation of crop growth. Initially, predefined crop growth recipes, established through biological experiments and references, are used as default settings in Ostara. These recipes include ideal environmental parameters for crop growth, such as lighting duration, temperature, humidity, nutrient levels, minimum growing time to harvest, and expected yield, which are adopted for production scheduling. Ostara and Witness (the digital layer) fetch real-time sensor data from the physical farm and compare actual parameters with default ones to manage the crop nutrition delivery system, including monitoring LED lighting patterns, delivering nutrients, and controlling irrigation schedules. With complete records of real-time data, the information layer continuously analyses the growth patterns against actual environmental factors and proactively suggests adjustments to farm operations.

Further investigation into the historical operations of the farm provides essential information for developing a DT-enhanced production scheduling strategy. The maximum growing capacity is limited to 48 trays. Six main crops and five major customers were identified from customer orders. Each type of crop seeds has a different minimum growing time to harvest and a conversion factor from seed weight to crop weight. Customer orders vary, with fluctuations of 10 to 20 percent. Under high demand, the farm priorities crop scheduling based on customer loyalty and profitability. Customers who place more consistent orders to profitable crops would receive higher priority.

## Mathematical model

The vertical farm operates with standard cultivation practices to balance resource utilisation with fluctuating demand patterns. It is equipped with vertically stacked growing trays, capable of supporting optimal growth for a variety of crop seeds (seeds in short). Each tray has a limited seed capacity, with control over planting density and distribution.

A diverse range of seeds is grown on the farm, each selected on the basis of its growth characteristics and market value. Each seed type has a specific growth cycle, initial weight, and yield potential for planning planting schedules. Growth cycles vary, with some requiring shorter periods and others needing more extended periods to reach maturity.

The yield of each seed type is influenced by growth factors (e.g., light, humidity, irrigation patterns). These factors determine how efficiently a seed converts into a harvestable crop after reaching its minimum growth time, with additional growth yielding further benefits under optimal conditions. The objective encompasses fulfilling demand promptly, maximising resource efficiency when capacity exceeds demand, and ensuring optimal tray utilisation to avoid downtime. Information about the indoor environment, the capacity of the farm, and the demand from the customer is integrated into decision-making. For instance, given the characteristics of indoor farming, environmental factors are adjusted to estimate growth cycles for adaptive production scheduling.

Model variables and parameters:

- $x_{i,t}$: Quantity of seed type $i$ planted at the beginning of period $t$ (kg).
- $h_{i,t}$: Duration (in days) that seed type $i$ is kept growing when planted in period $t$.
- $y_{i,t}$: Yield of seed type $i$ harvested at period $t$ (kg).
- $SW_i$: Initial seed weight for type $i$ (kg).
- $GT_i$: Minimum growing time for seed type $i$ (days).
- $CF_i$: Baseline conversion factor from seed to product for seed type $i$ at $GT_i$.
- $C_i$: Additional daily conversion factor per seed type $i$ post $GT_i$.
- $D_{i,t}$: Demand for seed type $i$ in period $t$ (kg).
- $B_i$: Benefit factor reflecting profitability and priority for seed type $i$.
- $E$: Electricity cost per day per kg of seed.
- $CAP$: Total capacity of growing trays.

where $i$, and $t$ represent the index for seed type, and time period respectively.

### MILP optimisation model

The objective function of the MILP optimisation approach aims to maximise resource efficiency and profitability while minimising the total mismatch between production and demand over all types of seeds throughout the planning period.

Objective functions: For scenarios where total demand is less than or equal to capacity, crops would be harvested after the minimum growing time since there is no urgency to free up the growing trays. In this situation, the objective is to optimise resource efficiency by minimising electricity usage while ensuring that demand is met:

$$\min \sum _{i,t} E \cdot x_{i,t} \cdot h_{i,t}$$

subject to the constraint that production fulfils the demand:

$$\sum _{t} y_{i,t} \ge D_{i,t} \quad \forall i$$

For scenarios where demand exceeds capacity, the objective is to maximise profitability adjusted for electricity costs: For scenarios where demand exceeds capacity, the objective is to maximise profitability adjusted for electricity costs, prioritising crops with higher benefit factors.

$$\max \sum _{i,t} (B_i \cdot y_{i,t} - \beta \cdot E \cdot x_{i,t} \cdot h_{i,t})$$

where $\beta$ is weighting factor for electricity costs.

Constraints:

1. Capacity constraint: Ensures that the total seeds planted at any time do not exceed the available capacity.

$$\sum _{i} x_{i,t} \le CAP \quad \forall t$$

2. Growth and yield constraints: Yield is calculated based on whether the seed is harvested after reaching its minimum growth time:

$$y_{i,t} = CF_i \cdot SW_i + C_i \cdot SW_i \cdot (h_{i,t} - GT_i)^\gamma \quad \text {if } h_{i,t} > GT_i$$

Here, $\gamma$ introduces nonlinearity, reflecting diminishing returns on additional growth time. Non-linearity is introduced through $\gamma$, which represents diminishing returns on extended growth time, as described in the yield equation. This is addressed using a piecewise-linear approximation to ensure computational tractability.

3. Harvesting time window: Ensure that all seeds are harvested within or at the end of the 4-week period:

$$t + GT_i \le 28 \cdot k + 28 \quad \text {for seed } i \text { planted at time } t \text { in cycle } k$$

This constraint allows strategic planting within each cycle to meet current or upcoming demand.

**Fig. 2:**
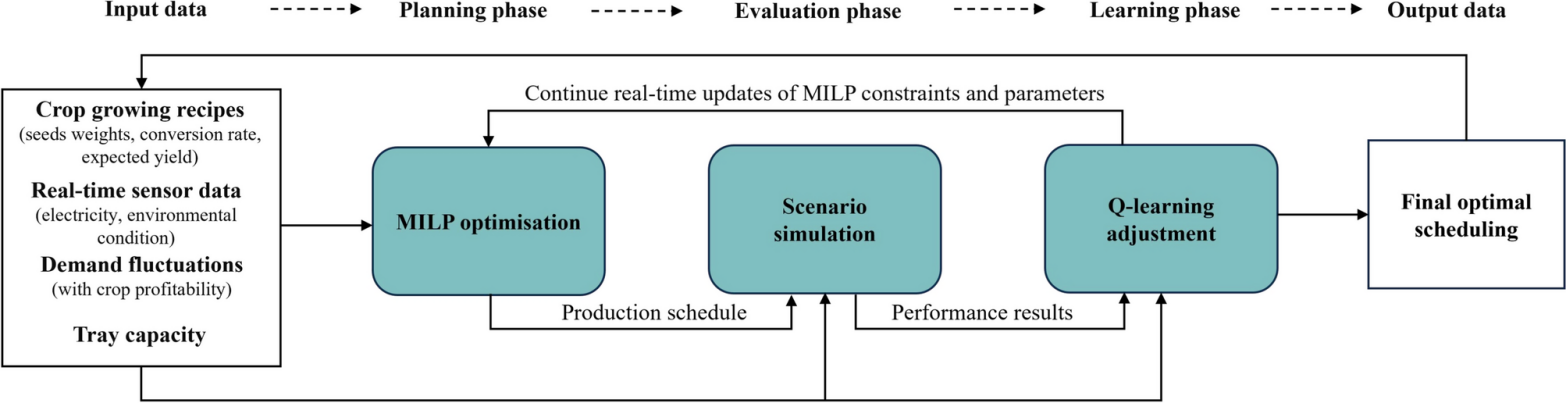
A flow chart of the MILP-Q-learning optimisation process.

### Q-learning for demand fulfillment optimisation

Q-learning is a reinforcement learning algorithm that estimates the optimal state-action value function, $Q(s, a)$, which represents the expected cumulative reward of taking action $a$ in state $s$ and following the optimal policy thereafter. The Q-function is defined as:

$$\begin{aligned} Q(s, a) = \mathbb {E} \left[ \sum _{t=0}^{\infty } \gamma ^t r_t \mid s_0 = s, a_0 = a \right] \end{aligned}$$

where $\gamma$ is the discount factor that balances the importance of immediate and future rewards, and $r_t$ is the reward received at time $t$.

The Q-learning update rule iteratively refines the Q-values based on the observed rewards and estimated future rewards, as follows:

$$\begin{aligned} Q_{new}(s, a) = Q(s, a) + \alpha \left[ r + \gamma \max _{a'} Q(s', a') - Q(s, a) \right] \end{aligned}$$

where $\alpha$ is the learning rate, $r$ is the immediate reward, $s'$ is the next state, and $a'$ is the action that maximises the Q-value in the next state.

In the context of demand fulfillment optimisation, states $s$ represent the current configuration of growing trays and the remaining demand for each customer, while actions $a$ correspond to planting specific crops in particular trays at designated time periods. The reward $r$ is designed to reflect the degree of demand fulfillment, incorporating both positive rewards for meeting demand and penalties for unmet demand or inefficient resource usage.

The reward function can be expressed as:

$$\begin{aligned} r = \sum _{c \in \text {customers}} \sum _{s \in \text {seeds}} \left( \min (D(s, c, t), p(s, c, t)) - \lambda \times \text {penalty terms} \right) \end{aligned}$$

where $D(s, c, t)$ is the demand for seed $s$ from customer $c$ at time $t$, $p(s, c, t)$ is the production or fulfillment of seed $s$ for customer $c$ at time $t$, and $\lambda$ is a weighting factor for penalty terms. The penalty terms can include various inefficiencies, such as the overuse of trays or planting seeds at suboptimal times. By incorporating these penalties, the reward function encourages the agent to make decisions that not only meet demand but also optimise resource utilisation.

Additionally, the reward function incorporates the benefit factor with consistency to prioritise crops based on their profitability and customer order consistency. The adjusted benefit factor $B_{s,c}$ is calculated as:

$$\begin{aligned} \overline{V}_{c} = \frac{1}{T} \sum _{t=1}^{T} V_{s,c,t} \end{aligned}$$

$$\begin{aligned} \kappa _c = \frac{\overline{V}_{c}}{\frac{1}{N} \sum _{c=1}^{N} \overline{V}_{c}} \end{aligned}$$

$$\begin{aligned} B_{s,c} = B_s \times (1 + \alpha \times \kappa _c) \end{aligned}$$

where $\overline{V}_{c}$ is the average order volume for customer $c$, $\kappa _c$ is the consistency of the customer’s orders, $B_s$ is the base benefit factor for crop type $s$, and $\alpha$ is a weighting factor for the consistency adjustment.

**Table 2:** variables and initial values.

Crop types	Growing time (days)	Seeds weight (kg)	Conversion factor	Customer 1 (kg)	Customer 2 (kg)	Customer 3 (kg)	Customer 4 (kg)	Customer 5 (kg)
Garlic chive	7	0.01	10	7	8.5	6.5	9	10.5
Sunflower	4	0.012	12	8	7.5	5.5	6.5	8.2
Radish	8	0.01	11	9.5	10	6	8.8	9.2
Coriander	6	0.015	14	7.8	6.5	5.2	6.9	5.8
Micro parsley	9	0.007	9	9	6	4.5	6.2	5.7
Basil	11	0.018	13	8.2	8.5	6	7.3	9.1

**Fig. 3:**
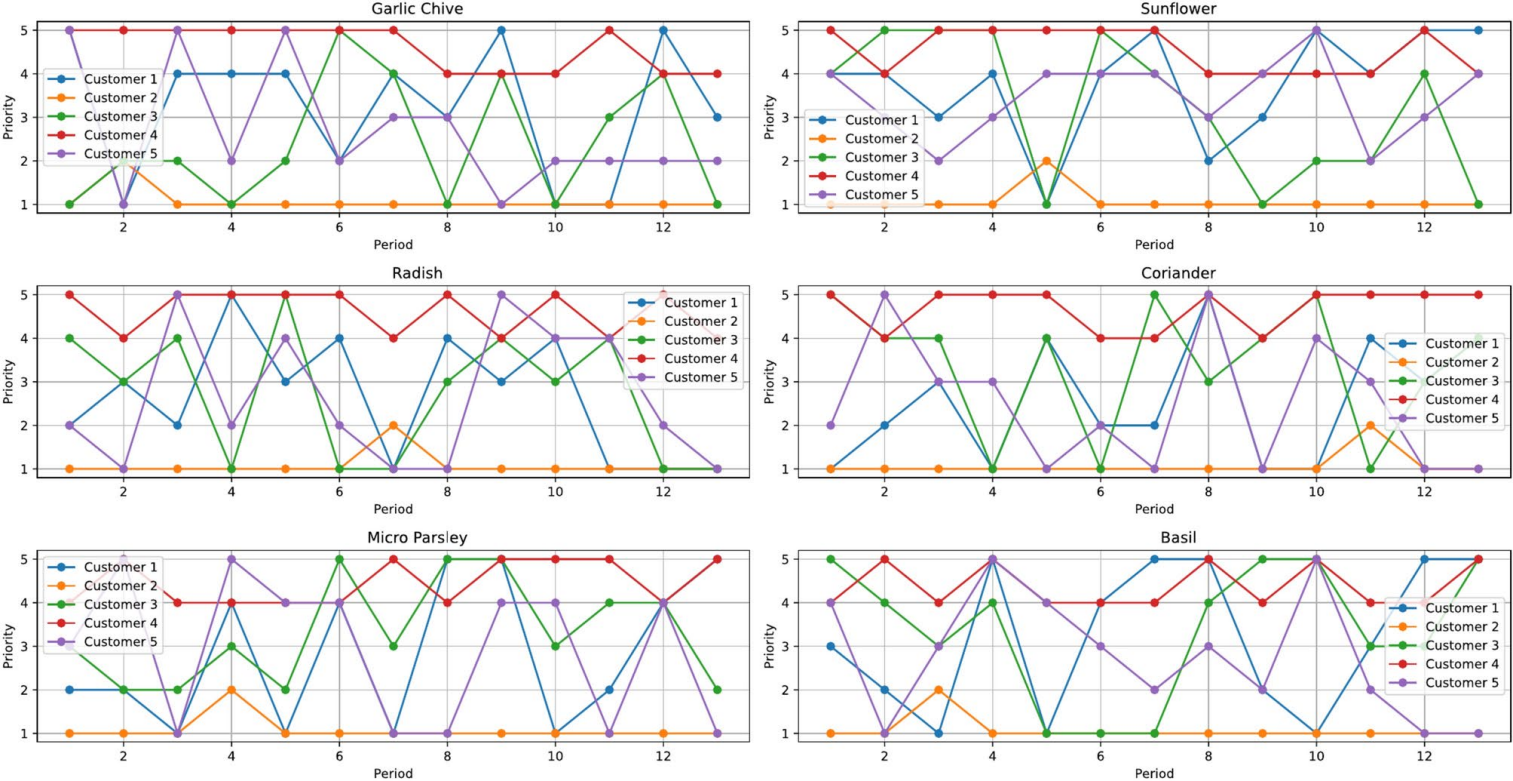
A sample distribution of customer priority by crop types.

During training, the Q-learning algorithm iteratively updates the Q-values based on the observed rewards and transitions. The training process involves initialising the Q-table, selecting actions based on an exploration-exploitation strategy (such as the $\epsilon$-greedy policy), executing actions, observing rewards, and updating Q-values. This process is repeated over multiple episodes until the Q-values converge, indicating that the agent has learned the optimal policy.

Mathematically, the Q-value update during training can be written as:

$$\begin{aligned} Q_{new}(s, a) = (1 - \alpha ) Q(s, a) + \alpha \left[ r + \gamma \max _{a'} Q(s', a') \right] \end{aligned}$$

where $Q(s, a)$ is the current Q-value, $r$ is the observed reward, $s'$ is the next state, and $\max _{a'} Q(s', a')$ is the maximum Q-value for the next state.

The demand fulfillment is then calculated as the sum of the fulfilled demands over the planning horizon, normalised to a scale of 0 to 100 for comparison purposes. The normalisation is done by dividing the achieved fulfillment by the maximum possible demand and scaling it by 100:

$$\begin{aligned} \text {Normalised demand fulfillment}(s, c) = \left( \frac{\text {Demand fulfillment}(s, c)}{\max (D(s, c, t))} \right) \times 100 \end{aligned}$$

where $\max (D(s, c, t))$ is the total demand for seed $s$ from the customer $c$ for all time periods.

By training the Q-learning model with this approach, the algorithm learns to make production decisions that maximise demand fulfillment while minimising inefficiencies. A flow chart in Figure 2 demonstrate the MILP-Q learning modelling process.

## Results

### Initial setting of variables and parameters

The farm investigation summarises the average monthly demand in kilograms from each customer, as shown in Table 2. The monthly demand fluctuates by +/- 10 to 20 percent of the previous period’s demand for each seed type and customer. The prioritisation of customers is ranked on a scale where a value of 1 indicates the highest priority and a value of 5 the lowest, based on the order consistency and profitability. Figure 3 presents an example of the priority distribution of customers with respect to each type of crop in a calendar year.

### Optimisation results

**Fig. 4:**
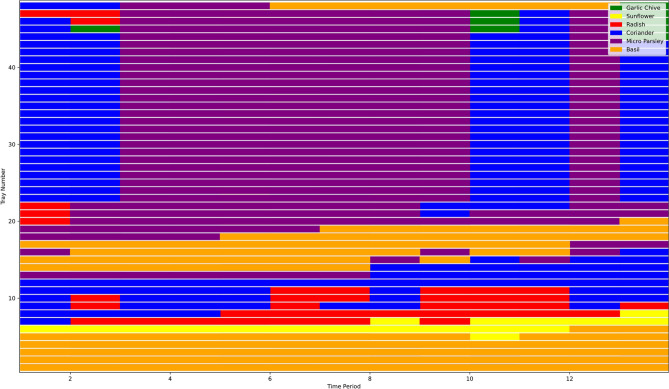
A sample of optimal planting and harvesting schedule.

Following the farm and crop settings of the case, the MILP optimisation model and the MILP-Q learning model optimise crop production scheduling over a calendar year, considering demand uncertainty and capacity constraints. A sample of the optimal planting and harvesting schedule is presented in Figure 4, which illustrates the planting and harvesting time, tray location and volume of crops for each crop on a monthly basis.

**Fig. 5:**
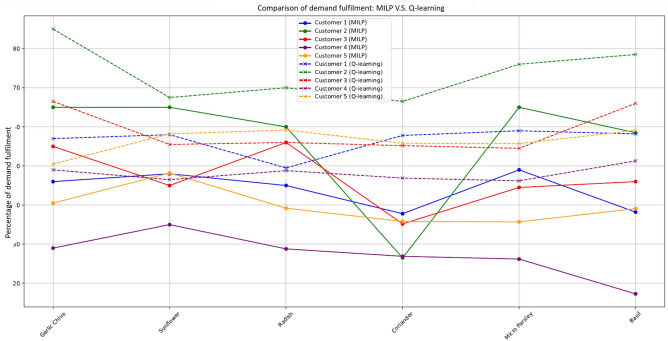
Demand fulfillment comparison by crop types: MILP V.S. Q-learning.

**Fig. 6:**
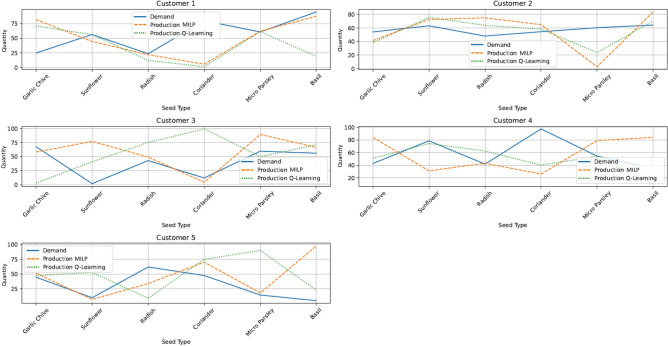
Difference between demand and production by crop types: the MILP V.S. Q-learning.

Given the varying priorities of crops and their respective customer demands, the Q-learning model outperforms the MILP optimisation model in terms of demand fulfillment across different crops. Figure 5 illustrates the satisfaction of demand for each seed type under two models: the MILP model (solid lines) and the Q-learning model (dashed lines).This figure compares performance across five different customers for each type of crop, clearly showing that the Q-learning model consistently achieves higher demand fulfillment than the MILP approach for all seed types across all customers. Figure 6 provides a detailed comparison of production and demand for each seed type (Garlic Chive, Sunflower, Radish, Coriander, Micro Parsley, Basil) across five different customers. It includes the actual demand (solid blue lines), production using the MILP model (dash orange lines), and production using the Q-learning model (dash green lines). In Figure 6, the MILP model shows a variation between demand and production, often falling short of meeting the exact demand due to capacity pressure, while the Q-learning model aligns more closely with the actual demand, indicating a better adaptation to demand uncertainty.

Table 3 provides a comprehensive comparison between the MILP and Q-learning models, focusing on production quantities and demand fulfillment rates across different crops and customers. This analysis takes into account variations in crop demand and electricity costs over 100 iterations. In contrast, Table 4 summarises the overall system performance of both models across 100 one-year iterations. The results consistently show that the Q-learning model achieves higher demand fulfillment rates than the MILP model across various demand scenarios. Overall, the Q-learning model demonstrates higher fulfillment rates than the MILP model. For example, for Garlic Chive (Customer 1), the fulfillment rate is 57.0 percent with Q-learning compared to 46.0 percent with MILP. Similarly, for Basil (Customer 2), the fulfillment rate is 78.5 percent with Q-learning compared to 58.5 percent with MILP. These results suggest that the Q-learning model is more effective in meeting customer demands, potentially leading to higher customer satisfaction and reduced stockouts. The Q-learning model consistently performs better in scenarios with varying demand, as evidenced by its higher and more consistent fulfillment rates across different customers and seed types. Additionally, while the operational costs are relatively similar between the two models, the Q-learning model often achieves higher fulfillment rates without significantly increasing electricity costs. The consistency of the Q-learning model in achieving higher fulfillment rates and effectively managing production towards demand fluctuations underscores its robustness and potential as a preferred method.

Q-learning model’s performance underscores the potential of DT-based optimisation in complex agricultural systems. From the perspective of DT configuration (see Figure 1, the Q-learning model functions as the analysis engine in the information layer, utilising the continuously updated farm data to perform adaptive learning. The learning results are then used to update the farm’s production decision-making processes, ensuring that the farm can dynamically adjust production plans to changing conditions and demands. The higher fulfillment rates achieved by the Q-learning model indicate its ability to adapt to varying demand scenarios, leveraging (near) real-time DT data for adaptive learning and decision-making. This adaptability is a critical feature of DTs, enabling them to provide robust solutions in dynamic environments. The consistent success of the Q-learning model in managing production and responding to demand fluctuations underscores the robustness and potential of DTs in urban and controlled environment agriculture.

**Table 3:** Accumulative comparison between MILP and Q-learning models over 100 iterations.

Crop	Customer	Demand	Production (MILP)	Production (Q_Learning)	Fulfillment rate (MILP)	Fulfillment rate (Q_Learning)	Electricity_Cost
Garlic chive	1	67	31	38.08	46.00	57.00	0.37
Garlic chive	2	92	60	78.46	65.00	85.00	0.35
Garlic chive	3	86	47	57.34	55.00	66.50	0.70
Garlic chive	4	95	27	46.39	29.00	49.00	0.57
Garlic chive	5	75	30	37.86	40.50	50.50	0.13
Sunflower	1	124	59	71.86	48.00	58.00	0.51
Sunflower	2	64	42	43.47	65.00	67.50	0.49
Sunflower	3	69	31	38.56	45.00	55.50	0.02
Sunflower	4	76	27	35.50	35.00	46.50	0.87
Sunflower	5	59	28	34.23	48.20	58.20	0.80
Radish	1	108	49	53.54	45.00	49.50	0.80
Radish	2	147	88	103.01	60.00	70.00	0.77
Radish	3	45	25	25.17	56.00	56.00	0.84
Radish	4	86	25	41.87	28.80	48.80	0.77
Radish	5	89	35	52.70	39.20	59.20	0.02
Coriander	1	102	39	59.21	37.80	57.80	0.09
Coriander	2	62	16	41.07	26.50	66.50	0.56
Coriander	3	55	19	30.31	35.20	55.20	1.00
Coriander	4	93	25	43.41	26.90	46.90	0.91
Coriander	5	68	24	37.90	35.80	55.80	0.02
Micro parsley	1	67	33	39.76	49.00	59.00	0.45
Micro parsley	2	122	79	92.50	65.00	76.00	0.60
Micro parsley	3	54	24	29.26	44.50	54.50	0.10
Micro parsley	4	96	25	44.51	26.20	46.20	0.74
Micro parsley	5	66	24	36.92	35.70	55.70	0.51
Basil	1	64	24	37.22	38.20	58.20	0.09
Basil	2	105	61	82.16	58.50	78.50	0.82
Basil	3	92	42	60.69	46.00	66.00	0.96
Basil	4	174	30	89.39	17.30	51.30	0.25
Basil	5	67	26	39.31	39.10	59.10	0.17

## Discussion

This research addresses the critical need for digital innovation in urban food production, focusing on the integration of DT technology in vertical farming, exemplified by the Grow It York case. We propose a proactive production strategy driven by DT data through MILP-Q learning modelling to optimise resource use and enhance decision-making processes. Our findings demonstrate that the integration of real-time historical production data into food production scheduling, complemented by a reward system in the Q-learning model for closed-loop feedback automation (see Figure 1), shows significant advantages for operational performance^20^^15^. Our findings demonstrate that the DT-enhanced scheduling model (the Q-learning model) maintains resilient demand fulfillment and resource utilisation under compound demand and priority uncertainties. This study sets a precedent for the integration of advanced DT technologies in urban food systems, emphasising production behaviour modelling and practical utility.

Our hybrid MILP-Q learning method offers a fresh take on tackling the challenges of urban vertical farming. By blending MILP’s knack for managing things like capacity and resources with Q-learning’s ability to handle uncertainties such as crop growth changes and fluctuating demand, we create a versatile solution. By integrating DT technology, we can continuously receive real-time data, which lets us keep optimising our processes on the go. This approach works really well in controlled environment agriculture because it relies on precise environmental settings and regular harvest cycles-perfect conditions for our reinforcement learning to do its job.

**Fig. 7:**
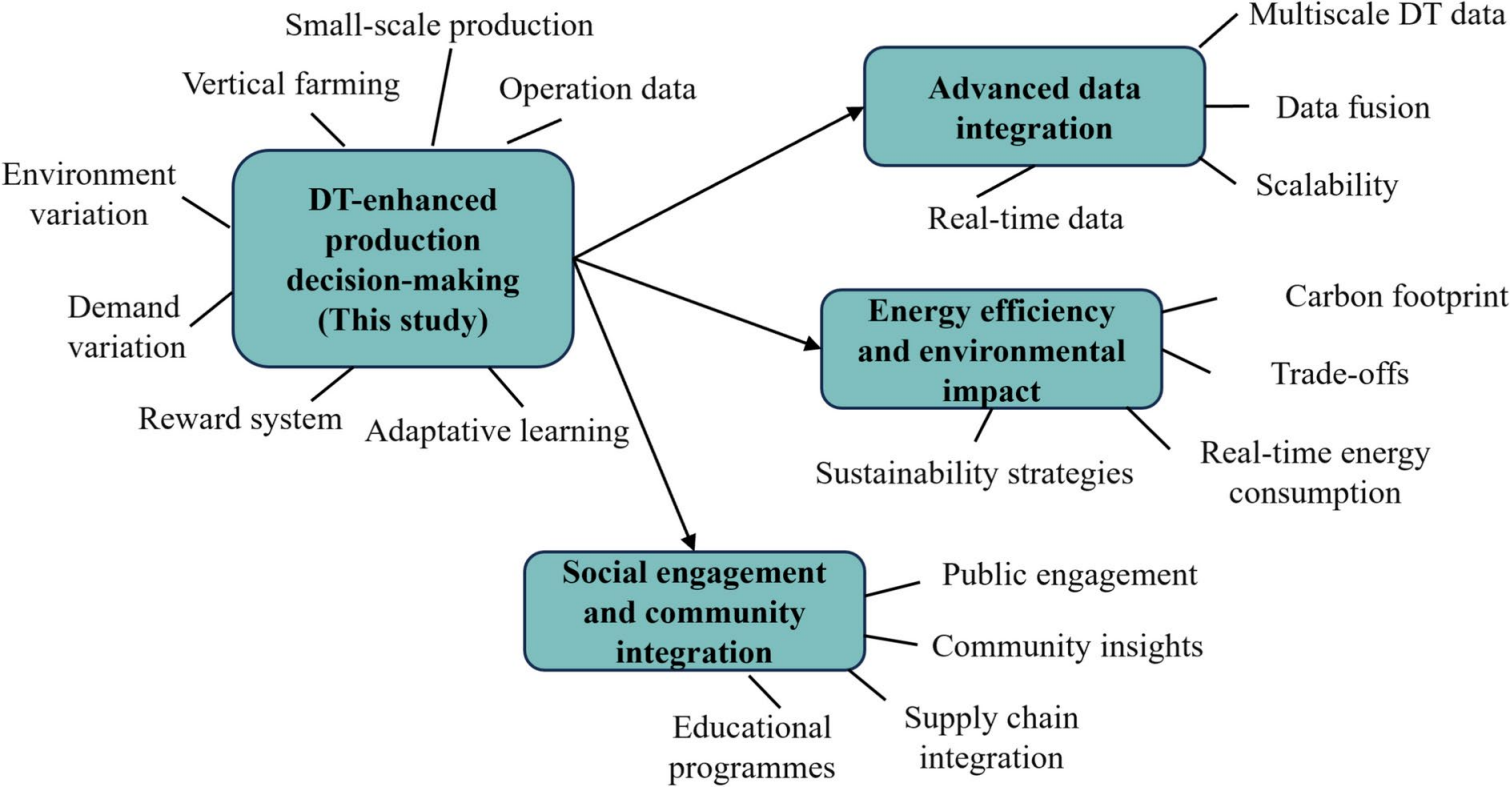
Future research streams.

Future research should further investigate real-time DT data integration approaches to enhance the scalability and efficiency of DT models in larger urban agricultural systems, considering multiscale DT data attributes, the environmental paradox, and social engagement. Below, we discuss these three future work streams in details (see Figure 7).

**Table 4:** Overall demand fulfillment rate of MILP and Q-learning model.

No.	Demand	FR (MILP)	FR (Q-Learning)	No.	Demand	FR (MILP)	FR (Q-Learning)	No.	Demand	FR (MILP)	FR (Q-Learning)
1	126	65.83%	72.55%	35	86	65.77%	72.05%	69	143	70.92%	78.45%
2	139	63.33%	68.41%	36	131	60.04%	66.41%	70	150	56.83%	63.60%
3	130	73.78%	80.72%	37	131	64.94%	71.24%	71	158	73.55%	80.45%
4	126	56.94%	64.79%	38	131	56.78%	63.40%	72	145	63.90%	69.91%
5	117	58.97%	63.96%	39	156	65.94%	72.55%	73	120	73.56%	81.38%
6	133	58.06%	64.37%	40	136	72.66%	78.38%	74	144	66.49%	73.07%
7	118	67.41%	73.78%	41	112	61.05%	68.64%	75	94	69.05%	74.74%
8	152	59.81%	66.10%	42	118	67.68%	73.15%	76	133	55.74%	62.17%
9	157	63.86%	68.75%	43	137	57.50%	63.25%	77	96	60.37%	64.45%
10	114	59.64%	67.46%	44	90	68.61%	76.18%	78	156	57.28%	62.49%
11	144	58.02%	63.81%	45	135	60.50%	67.72%	79	124	60.63%	67.27%
12	125	57.10%	64.48%	46	135	58.48%	65.30%	80	116	57.26%	62.42%
13	128	67.47%	74.27%	47	101	66.14%	70.54%	81	105	61.04%	67.51%
14	154	57.63%	62.82%	48	95	55.38%	63.06%	82	143	62.87%	68.59%
15	90	58.74%	65.99%	49	109	70.75%	77.61%	83	119	56.22%	60.76%
16	92	62.01%	67.59%	50	112	55.09%	63.08%	84	128	68.16%	73.35%
17	87	70.60%	78.12%	51	126	65.83%	72.55%	85	86	65.77%	72.05%
18	147	56.84%	63.17%	52	139	63.33%	68.41%	86	131	60.04%	66.41%
19	143	70.92%	78.45%	53	130	73.78%	80.72%	87	131	64.94%	71.24%
20	150	56.83%	63.60%	54	126	56.94%	64.79%	88	131	56.78%	63.40%
21	158	73.55%	80.45%	55	117	58.97%	63.96%	89	156	65.94%	72.55%
22	145	63.90%	69.91%	56	133	58.06%	64.37%	90	136	72.66%	78.38%
23	120	73.56%	81.38%	57	118	67.41%	73.78%	91	112	61.05%	68.64%
24	144	66.49%	73.07%	58	152	59.81%	66.10%	92	118	67.68%	73.15%
25	94	69.05%	74.74%	59	157	63.86%	68.75%	93	137	57.50%	63.25%
26	133	55.74%	62.17%	60	114	59.64%	67.46%	94	90	68.61%	76.18%
27	96	60.37%	64.45%	61	144	58.02%	63.81%	95	135	60.50%	67.72%
28	156	57.28%	62.49%	62	125	57.10%	64.48%	96	135	58.48%	65.30%
29	124	60.63%	67.27%	63	128	67.47%	74.27%	97	101	66.14%	70.54%
30	116	57.26%	62.42%	64	154	57.63%	62.82%	98	95	55.38%	63.06%
31	105	61.04%	67.51%	65	90	58.74%	65.99%	99	109	70.75%	77.61%
32	143	62.87%	68.59%	66	92	62.01%	67.59%	100	112	55.09%	63.08%
33	119	56.22%	60.76%	67	87	70.60%	78.12%				
34	128	68.16%	73.35%	68	147	56.84%	63.17%				

### Advanced real-time integration

Our results demonstrate that integrating data from farm operations, crop growth, demand variations, and priorities for adaptive production scheduling significantly enhances production decision-making in dynamic environments. The DT-enhanced scheduling model ensures that demand fulfillment and resource utilisation remain resilient to compound demand and priority uncertainties^20^^15^. This practical implementation of DTs showcases their utility in real-world settings, enhancing urban food production efficiency and resilience.

In comparison to the literature, our findings align with studies highlighting the benefits of DTs in industrial applications and extend this understanding to the context of urban food production. While previous research has focused on the theoretical potential of DTs in production and supply chains^14^, our study provides empirical evidence of their feasibility and effectiveness in streamlining operations of a real-world case, making production more flexible and efficient. This contributes to the body of knowledge by offering a replicable model for urban food production.

However, our study primarily focuses on production activities, including crop planting operations, demand variations, and farm conditions. Future research should explore specifics types of real-time farm data necessary for DTs, such as sensors for irrigation schedules, lighting data, temperature, and humidity. These additional data types could improve the accuracy of forecasting crop growth patterns and the conversion factor of seed to crop weight for real-time estimation. Therefore, there is a need for data fusion and model fusion considerations at the subsystem level of DTs.

### Environmental impact and energy efficiency

Urban vertical farms are energy-intensive due to the precise control required over the growing environment^1^. This setup significantly affects energy use. While DTs can potentially reduce this impact through precise control mechanisms, there is a paradox: urban vertical farming enables shorter food supply chains, reducing carbon emissions from delivery and transportation, yet the high energy costs of maintaining controlled environments are often underassessed compared to traditional farming methods^1^. The literature on the net environmental impact of digitalised urban food production is limited, indicating a significant gap^2^. Future research should assess and optimise DTs from a carbon perspective, examining the trade-offs between traditional and urban farming practices.

This study considers the energy costs associated with crop growing times in trays, which require controlled environments for lighting, irrigation, and temperature. Linking actual energy consumption to DT optimisation models is a crucial extension. By integrating real-time energy consumption data with farm operation data, we can obtain real-time energy consumption patterns for different crop types and growth stages, enabling adjustments to energy assumptions in mathematical models. Another critical consideration is comparing the benefits of using DTs for energy reduction-such as carbon footprint from shorter delivery distances and better production scheduling-with the energy consumption required to maintain farm operations and DT-oriented devices.

### Social engagement and community integration

This study does not yet consider social effects, though DT holds the potential for sustainable agricultural systems^21,22^. However, during the investigation of the Grow It York vertical farm, a hybrid business model emerged, incorporating food donations to food banks and public education and exhibitions to engage the community with environmental considerations.

Based on real-world practices, future work could combine social engagement with DT technologies to facilitate real-time integration across supply chain members and social entities, thereby enhancing social welfare. DTs extend multistage supply chains by connecting stakeholders through a unified digital platform^23,24^, enabling real-time sharing of farm operations for better decision-making and coordination. This also allows the public to engage in the process of digital farming.

Future research should consider DT data integration on a wider scale across business entities to support comprehensive cradle-to-grave sustainability analysis. Developing DT models informed by community insights and crafting social welfare strategies that utilise DT data to promote sustainable urban farming practices are essential pathways for future research. Engaging local communities in the development and implementation of DT-driven urban farming can enhance the social acceptance and effectiveness of these technologies.

## Conclusion

This research addresses the critical need to explore digital innovation in urban food production, focusing on the integration of DT in vertical farming decision making, as exemplified by the Grow It York case. This study proposes a proactive productions strategy driven by DT data - MILP-Q learning modelling- to optimise resource use and enhancing decision-making processes. The findings will set a precedent for the data integration of advanced DT technologies in urban food production systems focusing on production behaviour modelling. The results echo that the real-time integration of historical production activities feed into farm scheduling with a reward system designed for desired operational performance (i.e., demand fulfillment or resource efficiency) can enhance production efficiency and resilience towards demand and production uncertainties. In conclusion, our research highlights the transformative potential of DT-based optimisation in urban food production. By integrating real-time data collection and adaptive learning through Q-learning, we demonstrate the practical utility and scalability of DTs. Future research should continue to explore these themes, focusing on energy efficiency, multiscale data fusion, and community engagement to advance the field of digital regenerative food systems.

## Data Availability

The datasets used and analysed during the current study are available from the corresponding author on reasonable request.

## References

[1] Van Delden, S. et al. Current status and future challenges in implementing and upscaling vertical farming systems. *Nat. Food.* **2** (2021). 10.1038/s43016-021-00402-w 37118238

[2] Ghandar, A. et al. A decision support system for urban agriculture using digital twin: A case study with aquaponics. *IEEE Access*. **9**, 35691–35708 (2021).

[3] Tao, F., Zhang, M., Liu, Y. & Nee, A. Y. C. Digital twin in industry: State-of-the-art. *IEEE Trans. Industr. Inform.*. **15**, 2405–2415. 10.1109/TII.2018.2873186 (2018).

[4] Huang, Y., Ghadge, A. & Yates, N. Implementation of digital twins in the food supply chain: a review and conceptual framework. *Int. J. Prod. Res.*. 1–27 (2024).

[5] Badakhshan, E. & Ball, P. Deploying hybrid modelling to support the development of a digital twin for supply chain master planning under disruptions. *Int. J. Prod. Res.* **62**, 3606–3637. 10.1080/00207543.2023.2244604 (2024).

[6] Guidani, B., Ronzoni, M. & Accorsi, R. Virtual agri-food supply chains: A holistic digital twin for sustainable food ecosystem design, control and transparency. *Sustain. Prod. Consum.* (2024).

[7] Singh, G., Rajesh, R., Daultani, Y. & Misra, S. Resilience and sustainability enhancements in food supply chains using digital twin technology: A grey causal modelling (gcm) approach. *Comput. Ind. Eng.* **179**, 109172 (2023).

[8] Ivanov, D. Conceptualisation of a 7-element digital twin framework in supply chain and operations management. *Int. J. Prod. Res.* **62**, 2220–2232 (2024).

[9] Kampker, A., Stich, V., Jussen, P., Moser, B. & Kuntz, J. Business models for industrial smart services - the example of a digital twin for a product-service-system for potato harvesting. *Procedia CIRP*. **83**, 534–540 (2019).

[10] Skobelev, P. et al. Development of models and methods for creating a digital twin of plant within the cyber-physical system for precision farming management. In *J. Phys. Conf. Ser.*, vol. 1703, 012022 (IOP Publishing, 2020).

[11] Laryukhin, V. et al. The multi-agent approach for developing a cyber-physical system for managing precise farms with digital twins of plants. *Cybern. Phys.* **8**, 257–261 (2019).

[12] Kim, S. & Heo, S. An agricultural digital twin for mandarins demonstrates the potential for individualized agriculture. *Nat. Commun.* **15**, 1561. 10.1038/s41467-024-45725-x (2024). 38378798 10.1038/s41467-024-45725-x PMC10879191

[13] Corsini, R., Costa, A., Fichera, S. & Framinan, J. Digital twin model with machine learning and optimization for resilient production-distribution systems under disruptions. *Comput. Ind. Eng.* 110145, 10.1016/j.cie.2023.110145 (2024).

[14] Li, L., Li, X., Chong, C., Wang, C. & Wang, X. A decision support framework for the design and operation of sustainable urban farming systems. *J. Clean. Prod.* **268**, 121928 (2020).

[15] Maheshwari, P., Kamble, S., Belhadi, A., Venkatesh, M. & Abedin, M. Digital twin-driven real-time planning, monitoring, and controlling in food supply chains. *Technol. Forecast. Soc. Change.* **195**, 122799 (2023).

[16] Mnih, V. et al. Human-level control through deep reinforcement learning. *Nature*. **518**, 529–533 (2015). 25719670 10.1038/nature14236

[17] Du, S., Zhou, W., Fei, M., Nee, A. & Ong, S. Bi-objective scheduling for energy-efficient distributed assembly blocking flow shop. *CIRP Annals*. (2024).

[18] York, G. I. Let’s grow it together. https://www.growityork.org/ (2024). Accessed April 16, 2024.

[19] Grow, L. Indoor farming for a better future. https://www.lettusgrow.com/ (2024). Accessed April 16, 2024.

[20] Longo, F., Mirabelli, G., Padovano, A. & Solina, V. The digital supply chain twin paradigm for enhancing resilience and sustainability against covid-like crises. *Procedia Comput. Sci.* **217**, 1940–1947 (2023). 36687282 10.1016/j.procs.2022.12.394 PMC9836493

[21] Basso, B. & Antle, J. Digital agriculture to design sustainable agricultural systems. *Nat. Sustain.* **3**, 254–256 (2020).

[22] Cesco, S. et al. Smart agriculture and digital twins: Applications and challenges in a vision of sustainability. *Eur. J. Agron.* **146**, 126809 (2023).

[23] Oehlschläger, D., Glas, A. & Eßig, M. Acceptance of digital twins of customer demands for supply chain optimisation: an analysis of three hierarchical digital twin levels. *Ind. Manag. Data Syst.* **124**, 1050–1075 (2024).

[24] Ivanov, D. Conceptualisation of a 7-element digital twin framework in supply chain and operations management. *Int. J. Prod. Res.* **62**, 2220–2232 (2024).

